# Hypocalcemia Revealing an Enteropathy-Associated T-cell Lymphoma

**DOI:** 10.22088/cjim.10.1.111

**Published:** 2019

**Authors:** Maria Belen Alonso Ortiz, Noel Lorenzo Villalba, Emilio Almaraz Marroquin, Melek Kechida, Manuel Mendez-Bailon, Imanol Pulido Gonzalez

**Affiliations:** 1 Department of Internal Medicine, Gran Canaria University Hospital, Las Palmas, Spain; 2 Department of Internal Medicine, Chretien Hospital, Liege, Belgium; 3 Department of Internal Medicine, Yeovil District Hospital, Yeovil, United Kingdom; 4 Department of Internal Medicine. Bourguiba University Hospital Fattouma, Monastir, Tunisia; 5 Department of Internal Medicine, San Carlos University Hospital, Madrid, Spain; 6 Department of Internal Medicine, Gran Canaria University Hospital, Las Palmas, Spain

**Keywords:** Hypocalcemia, Hypomagnesemia, Celiac disease, Enteropathy-associated T-cell lymphoma

## Abstract

**Background:**

Enteropathy-associated T-cell lymphoma (EATL) is a rare and aggressive type of extranodal T-cell lymphoma (TCL) arising in the gastrointestinal (GI) tract and represents 5–8% of all T-cell non-Hodgkin lymphomas (NHL) and 10–25% of primary intestinal lymphomas.

**Case presentation:**

We reported a 78-year-old woman presenting with severe hypocalcemia. Investigations confirmed vitamin D and iron deficiency as well as hypoalbuminemia. Celiac disease was suspected and confirmed, but despite intravenous calcium and magnesium supplementation and a gluten-free diet, normal electrolyte levels were never reached. Intestinal perforation was the clue to the diagnosis of enteropathy-associated T-cell lymphoma (EATL).

**Conclusion:**

Hypocalcemia can result from multiple conditions. In patients not responding to adequate supplementation, further investigations should be performed to diagnose the underlying condition

Hypocalcemia is an electrolyte disorder commonly encountered on medical and surgical services. While low serum calcium concentrations are often caused by disorders of parathormone (PTH) or vitamin D, this condition may result from a wide spectrum of clinical conditions. Therefore, full investigations are required to identify the underlying cause, particularly in patients not responding to adequate supplementation. Hypocalcemia can also be seen in malignant diseases (1, 2). A large variety of different and intriguing mechanisms leading to hypocalcemia may be involved but their full spectrum is not widely recognized. It has been suggested that this metabolic disorder occurs via mechanisms pertaining to osteoblastic-stimulating factors or toxic effects of certain chemotherapeutic agents (3). We reported a 78-year-old woman presenting with severe hypocalcemia in the setting of enteropathy-associated T-cell lymphoma. 

## Case Presentation

A 78-year-old woman presented to the emergency department complaining of paresthesias in her upper limbs and inability to open her hands over the last four hours. The patient experienced weight loss of about 14 kg over the previous 2 months, loss of appetite, and two to three liquid stools per day over the last 3 days.

Her medical history revealed a long history of hypertension, osteoporosis. Family history was noncontributory. Her current medications included bisoprolol 2.5 mg daily, calcium/vitamin D3 supplementation, and denosumab 60 mg every 6 months. Initial evaluation in the emergency department revealed marked hypocalcemia with a total calcium 6.0 mg/dL (normal 8.2 to 10.50 mg/dL) and ionic calcium 0.97 mmol/L (normal 1.15 to 1.35 mmol/L), hypomagnesemia (0.56 mg/dL), and normal phosphorus, renal function, white blood count, and hemoglobin. The chest x-ray and the electrocardiogram were normal. She was transferred to the Department of Internal Medicine. On physical exam, the patient looked ill. Blood pressure, heart rate, and oxygen saturation were normal. 

The cardiorespiratory system examination was normal, as well as the abdominal exam. Neurological exam was normal but she complained of paresthesias. The patient’s laboratory testing revealed no other electrolyte disorders, normal liver function, and normal blood gases. Her folic acid, vitamin D, and serum iron levels were low: 2.7 ug/L (normal: 5 to 15 µg/l), 14 ng/mL (normal: 30 to 50 ng/mL) and 18 ug/mL (normal: 25 to 150 ng/mL), respectively. A normochromic normocytic anemia was found. Albumin and total proteins were also low. Parathormone (PTH) was 452 pg/mL (normal: 0.00 to 68.2 pg/mL). Her thyroid function, lipid panel, and vitamin B12 levels were normal, and sedimentation rate was within the normal range. Autoimmune panel revealed positivity for anti-gliadin IgG antibodies and negativity for anti-transglutaminase and anti-endomysium antibodies. Anti-*Saccharomyces cerevisiae* antibodies (ASCA) antibodies were negative. The genetic studies revealed positivity for DR3DQ2 and DR4DQ8 haplotypes. Fecal chymotrypsin, calprotectin, and antitrypsin had increased. 

The patient underwent an upper endoscopy revealing the presence of villous atrophy in the proximal and middle small intestine without ulcers, findings that were consistent with celiac disease. The first abdominal computed tomography (CT) was read as negative. The patient was started on a gluten-free diet and intravenous calcium and magnesium supplementation with good clinical response. One week after admission the patient developed upper right quadrant pain and fever consistent with acute cholecystitis, confirmed by ultrasound, and she was started on intravenous antibiotics. Five days later, she developed edema in the left lower limb and a positive D-dimer. Venous doppler ultrasound and computed tomography of the chest confirmed the presence of deep venous thrombosis and pulmonary thromboembolism. Anticoagulation with low molecular weight heparin was initiated. There was no evidence of right ventricular dysfunction on the echocardiogram. 

On day 27 of admission the patient complained of acute abdominal pain. The abdominal computed tomography was consistent with intestinal perforation of uncertain location. The computed tomography also revealed the presence of abdominal adenopathy. She underwent resection of the distal jejunum and ileum. The anatomical and pathological study of the intestinal tissue showed transmural and multifocal infiltration (three lesions of about 3.2 cm in maximum diameter) with intestinal T-cell lymphoma with histopathological features compatible with T-cell lymphoma-associated with enteropathy. 

There were also signs of fibrinopurulent peritonitis. After surgery, the patient was transferred to the intensive care unit for the management of septic shock. She received 13 days of intravenous antibiotics with good clinical response. She was readmitted to the internal medicine department. The immunophenotype showed positivity for CD3, CD7, and CD30, partial positivity for CD2, CD4, and TIA1, and negativity for CD20, CD5, CD8, and CD56. Ki-67 was elevated consistently with a high proliferative index. Intravenous calcium and magnesium supplementation were eventually switched to oral supplementation and oral feeding was started. The patient refused oral anticoagulation and treatment for the lymphoma. She was discharged from the hospital 3 months after admission. 

## Discussion

Parathyroid hormone (PTH), vitamin D, calcium ion itself, and phosphate are the primary physiologic factors influencing serum calcium concentration (1). Parathyroid hormone and vitamin D disturbances are the most common causes of hypocalcemia. The metabolic panel of our patient showed hypocalcemia, low vitamin D and magnesium levels, and high PTH levels. It is likely that her PTH increased to compensate for a low serum calcium concentration, increasing reabsorption of calcium from the kidneys and bones and increasing production of 1,25-dihydroxyvitamin D. In addition, hypomagnesemia may increase PTH levels by parathyroid hormone (PTH) resistance (1, 2).

Another factor that may have contributed to hypocalcemia in this patient was treatment with denosumab. This is a human monoclonal antibody to the receptor activator of nuclear factor κB ligand (RANKL), leading to inhibition of osteoclast formation, decreasing bone resorption, and increasing bone mineral density (BMD) (3, 4, 5). In patients receiving this drug, underlying medical conditions such as vitamin D deficiency and malabsorption syndromes may predispose to the development of hypocalcemia as seen in our patient. Some reports have suggested hypocalcemia may arise after the first dose of denosumab with a median time from drug administration to calcium nadir of 25 days (ranging from 14 to 106 days) and median time to recover baseline calcium of 17 days (ranging from 6 to 40 days) (6). In the current case, our patient had received two doses with the last one being 3 months prior to hospitalization. Further testing was performed to determine why calcium levels remained low despite supplementation. Laboratory results showed decreased albumin and iron deficiency, suggesting malabsorption which was confirmed through the exams performed. Nevertheless, despite a gluten-free diet, IV calcium, and magnesium supplementation as well as vitamin D supplementation, optimal levels were never reached.

The intestinal perforation was the key factor in the discovery of the underlying disease of T-celllymphoma. Enteropathy-associated T-cell lymphoma (EATL) is a rare and aggressive type of extranodal T-cell lymphoma (TCL) arising in the gastrointestinal (GI) tract and represents 5–8% of all T-cell non-Hodgkin lymphomas (NHL) and 10–25% of primary intestinal lymphomas (7, 8). It is most commonly seen in men (in contrast to our patient) between the sixth and seventh decades of life. Type I EATL has been strongly associated with HLA-DQ2/DQ8 genotype and celiac disease (9). Gastrointestinal symptoms such as diarrhea, abdominal pain, and B symptoms are often the initial presentation. In contrast to the case, hypercalcemia is most commonly seen, reaching up to 91% in one of the series published (7). In the case presented, diarrhea developed early prior to admission and there was no previous history of celiac disease, but she did have a history of weight loss. Intestinal obstruction and perforation, as seen in this case, occur frequently in these patients. Studies are unclear about a beneficial effect of adherence to a gluten-free diet on EATL risk reduction (7). It is likely that T-cell lymphoma was already established in our patient by the time she developed her symptoms. Like most T-cell lymphoma subtypes, there is no standard of care for newly diagnosed EATL. However, CHOP-based chemotherapy with or without consolidative autologous stem cell transplantation (ASCT) remains the mainstay of treatment (10).

In conclusions** h**ypocalcemia can result from multiple conditions. In patients not responding to adequate supplementation, further investigations should be performed to diagnose the underlying condition 
